# Cytomegalovirus colitis after systemic chemotherapy in a patient with recurrent colon cancer: A case report

**DOI:** 10.1186/1752-1947-2-289

**Published:** 2008-08-28

**Authors:** Fuminori Teraishi, Hiroshi Shimamura, Takeo Suzuki, Masako Nakamoto, Akira Chikuba, Masashi Nezu, Shun Kohsaka, Takao Takiue, Hiroshi Chikuba

**Affiliations:** 1 Chikuba Hospital for Gastrointestinal and Colorectal Surgery, Kurashiki 710-0142, Japan; 2 Division of Cardiology, Columbia University, College of Physicians and Surgeons, New York, NY 10032, USA

## Abstract

**Introduction:**

The occurrence of cytomegalovirus colitis is well known in immunosuppressed patients, such as neoplastic patients following chemotherapy, although its exact etiology remains unclear.

**Case presentation:**

We present a case of cytomegalovirus colitis occurring in a 77-year-old man with vomiting and diarrhea 2 weeks after initial systemic chemotherapy consisting of 5-fluorouracil, leucovorin and irinotecan for a recurrent colorectal cancer. Initial colonoscopy revealed multiple punched-out ulcers in the transverse colon and the diagnosis of cytomegalovirus was based on positive cytomegalovirus antigen detected by indirect enzyme antibody method, although immunohistological examination of tissues biopsied at colonoscopy was negative. The symptoms ceased under ganciclovir and octreotide treatment, and the patient recovered gradually.

**Conclusion:**

The most probable cause of the cytomegalovirus colitis in this case was impaired immunity following chemotherapy. Cytomegalovirus infection should be included in the differential diagnosis of gastrointestinal disease in colorectal cancer patients after chemotherapy and, when suspected, the clinician should pursue appropriate diagnostic interventions including colonoscopy.

## Introduction

Recently, a number of new treatment options have become available for colorectal cancer (CRC) patients. In particular, CPT-11 (irinotecan), a specific inhibitor of topoisomerase I, has been proven to have efficacy in the treatment of CRC. Most recent studies have demonstrated a significant improvement in the addition of irinotecan to 5-fluorouracil (5-FU)-leucovorin (LV) combination therapy (FOLFIRI) for patients with 5-FU-refractory advanced CRC. In contrast, immunosuppression induced by chemotherapy is less well characterized, with opportunistic infections appearing mainly after high-dose treatment or with certain new drugs directly affecting lymphocyte homeostasis. The occurrence of cytomegalovirus (CMV) colitis is well known in immunosuppressed patients, such as neoplastic patients after chemotherapy, although its exact etiology remains unclear. We describe the case of a 77-year-old man presenting with an unusual viral complication with CMV colitis diagnosed 2 weeks after a first course of standard chemotherapy for a recurrent CRC.

## Case presentation

A 77-year-old man with known recurrence of colon cancer, which was not previously treated with any adjuvant chemotherapies, was admitted for vomiting and diarrhea 2 weeks after standard FOLFIRI chemotherapy consisting of 5-FU (350 mg/bolus/m^2 ^plus 2300 mg/infuser pump/m^2^), LV (200 mg/m^2^) and irinotecan (130 mg/m^2^). Laboratory examination on admission showed a white blood cell count of 15,300/mcl and C-reactive protein of 16.4 mg/dl, and he was also slightly anemic. Blood, urine and stool cultures as well as Clostridium difficile toxin 33 assay for stool specimen results were negative.

**Figure 1:**
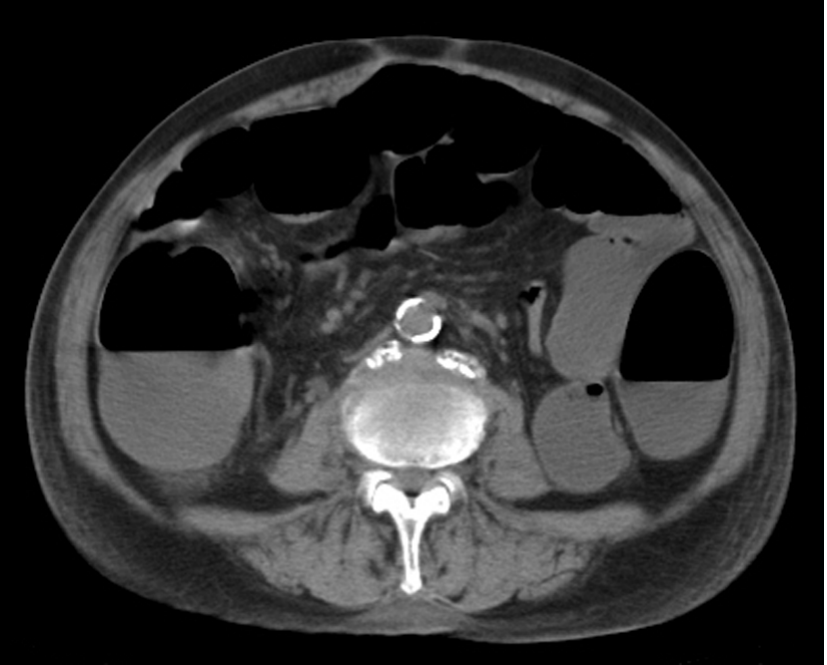
Abdominal computed tomography shows massive large bowel dilatation of the entire colon.

**Figure 2:**
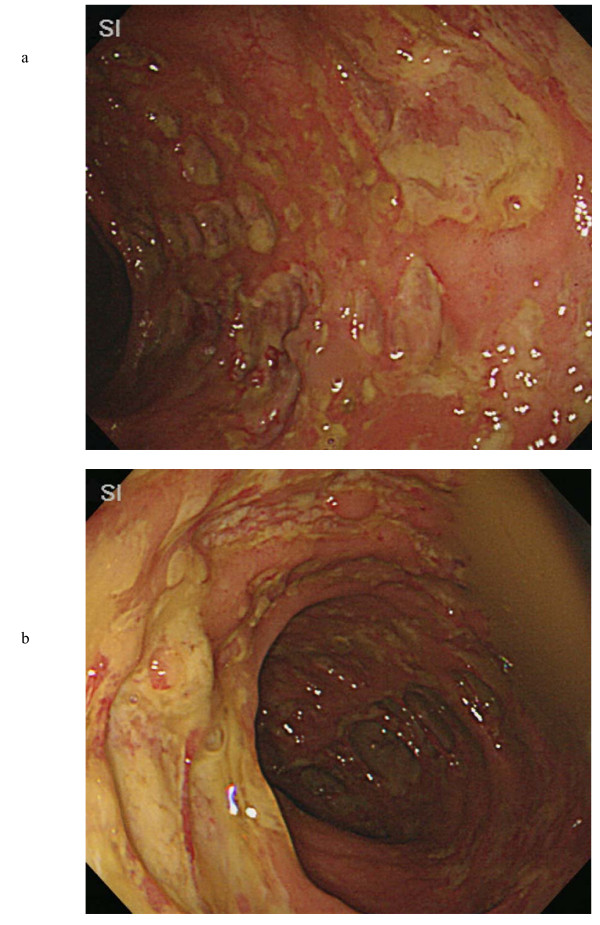
Initial colonoscopy reveals multiple punched-out ulcers of the right transverse colon (A) and the left transverse colon (B) with minimal granularity in the surrounding mucosa.

**Figure 3:**
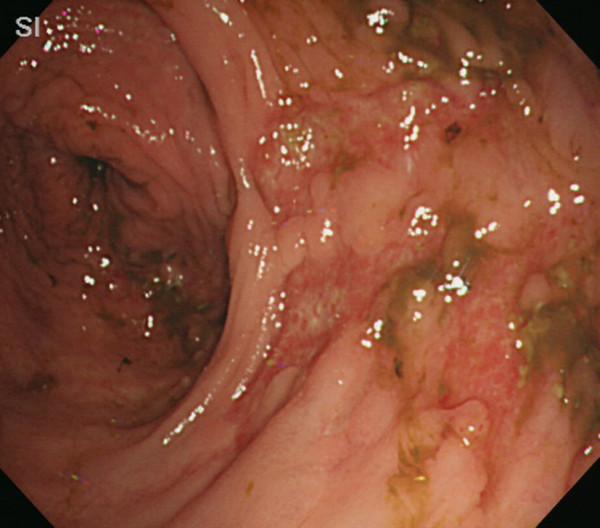
Follow-up colonoscopy demonstrates partial healing of the ulcers in the transverse colon.

Abdominal computed tomography imaging revealed a massive dilatation of the entire colon (Figure 1). Subsequently, a colonoscopy was performed, which revealed multiple punched-out ulcers in the transverse colon (Figure 2A and 2B) typical for CMV colitis. Following colonoscopy, CMV antigen was detected by indirect enzyme antibody method, also known as antigenemia method, but the biopsy specimens did not reveal CMV inclusion body immunohistologically. Based on these findings, the patient was diagnosed with CMV colitis and was started on intravenous ganciclovir therapy (500 mg/day for 2 weeks) combined with subcutaneous octreotide (200 mcg/day for 10 days). The patient gradually improved, and a second colonoscopy 4 weeks after admission demonstrated partial healing of multiple ulcers in the transverse colon (Figure 3).

## Discussion

Although CMV colitis is not frequently encountered, severe CMV colitis has been reported in neoplastic patients after chemotherapy [1,2]. The diagnosis of CMV colitis often poses a clinical challenge. Although endoscopic biopsies taken from the mucosa and ulcer bed are a relatively rapid and reliable method for demonstrating CMV colitis, their sensitivity is sometimes limited [3]. CMV antigen in the blood may also confirm the diagnosis of CMV colitis [1] as in this case but may not always be present. Furthermore, the clinical symptoms of CMV colitis are indistinguishable from irinotecan-induced enteritis, another infrequently seen but important cause of severe colitis [4,5].

In our case, since the CMV antigen was positive, it is unlikely that the patient's colitis was directly related to the use of irinotecan, but the high-dose irinotecan may have been the major predisposing factor for the activation of CMV. Moreover, although it has been reported that irinotecan has the potential to induce neutropenia [5], our case showed an elevation of the leukocyte count, presumably caused by the colitis itself and the micro-abscesses.

The primary therapy for CMV colitis is the use of antiviral drugs such as ganciclovir. In addition, previous reports have shown that octreotide also has potential for use against CMV colitis, although its mechanism of action remains unclear [2,6]. Our case demonstrated a favorable response to these treatments. Despite successful treatment, elective surgery is warranted if intestinal stenosis develops after the punched-out ulcer has healed [7]. Although the overall frequency of CMV infection in patients with neoplasm after chemotherapy is uncertain, CMV colitis should be ruled out with colonoscopy if the patient is suspected of having atypical enteritis after chemotherapy, as demonstrated in our case.

As the clinical pathological features of CMV colitis and inflammatory bowel disease often overlap, and because of the possible co-existence of CMV colitis with idiopathic colitis, the possibility of CMV infection should always be considered, so that the most appropriate therapy can be instituted for these patients.

## Conclusion

This case demonstrates that CMV colitis appears to be an extremely rare but potentially serious complication for patients with CRC following chemotherapy. Therefore, in individuals with CRC who do not respond to traditional medical therapy, other diagnoses including CMV should be considered, with early examinations of colonoscopy with biopsy and CMV antigenemia.

## Abbreviations

CMV: cytomegalovirus; CRC: colorectal cancer; FOLFIRI: folinic acid (leucovorin) fluorouracil (5-FU) irinotecan (CPT-11); LV: leucovorin; 5-FU: 5-fluorouracil.

## Competing interests

The authors declare that they have no competing interests.

## Consent

Written informed consent was obtained from the patient for publication of this case report and accompanying images. A copy of the written consent is available for review by the Editor-in-Chief of this journal.

## Authors' contributions

FT summarized the case and designed and drafted the manuscript. HS, TT, and HC participated in the design and coordination of the manuscript. SK initialized the case report and helped to prepare the manuscript. TS, MN, AC, and MN read and approved the final manuscript.
